# Enablers and determinants of the provision of written action plans to patients with asthma: a stratified survey of Canadian physicians

**DOI:** 10.1038/s41533-017-0012-3

**Published:** 2017-03-31

**Authors:** Fabienne Djandji, Alexandrine J. Lamontagne, Lucie Blais, Simon L. Bacon, Pierre Ernst, Roland Grad, Kim L. Lavoie, Martha L. McKinney, Eve Desplats, Francine M. Ducharme

**Affiliations:** 1 Clinical Research and Knowledge Transfer Unit on Childhood Asthma, Research Centre, CHU Sainte-Justine, Montreal, Quebec Canada; 2 Department of Family Medicine, McGill University, Montreal, Quebec Canada; 3 Faculty of Pharmacy, University of Montreal, Montreal, Quebec Canada; 4 Department of Exercise Science, Concordia University, Montreal, Quebec Canada; 5 Montreal Behavioural Medicine Centre, CIUSS-NIM, Hopital du Sacré-Coeur de Montreal, Montreal, Quebec Canada; 6 Department of Pulmonary Medicine, Jewish General Hospital, Montreal, Quebec Canada; 7 Division of Clinical Epidemiology (MUHC) Epidemiology, Biostatistics and Occupational Health, McGill University, Montreal, Quebec Canada; 8 Department of Psychology, Université du Québec à Montréal, Montreal, Quebec Canada; 9 Department of Pediatrics, University of Montreal, Montreal, Quebec Canada; 10 Applied Clinical Research Unit, Research Centre, CHU Sainte-Justine, Montreal, Quebec Canada; 11 Department of Social and Preventive Medicine, University of Montreal, Montreal, Quebec Canada

## Abstract

Despite national recommendations, most patients with asthma are not given a written action plan . The objectives were to ascertain physicians’ endorsement of potential enablers to providing a written action plan, and the determinants and proportion, of physician-reported use of a written action plan. We surveyed 838 family physicians, paediatricians, and emergency physicians in Quebec*.* The mailed questionnaire comprised 102 questions on asthma management, 11 of which pertained to written action plan and promising enablers. Physicians also selected a case vignette that best corresponded to their practice and reported their management. The survey was completed by 421 (56%) physicians (250 family physicians, 115 paediatricians and 56 emergency physicians); 43 (5.2%) reported providing a written action plan to ≥70% of their asthmatic patients and 126 (30%) would have used a written action plan in the selected vignette. Most (>60%) physicians highly endorsed the following enablers: patients requesting a written action plan, adding a blank written action plan to the chart, receiving a copy of the written action plan completed by a consultant, receiving a monetary compensation for its completion, and having another healthcare professional explain the completed written action plan to patients. Four determinants were significantly associated with providing a written action plan: being a paediatrician (RR:2.1), treating a child (RR:2.0), aiming for long-term asthma control (RR:2.5), and being aware of national recommendations to provide a written action plan to asthmatic patients (RR:2.9). A small minority of Quebec physicians reported providing a written action plan to most of their patients, revealing a huge care gap. Several enablers to improve uptake, highly endorsed by physicians, should be prioritised in future implementation efforts.

## Asthma: Encouraging doctors to provide written action plans

Changes to practice organization and doctors’ perceptions should encourage the provision of written action plans for all asthma patients. International guidelines state that effective long-term treatment of asthma requires educated self-management, regular reviews and provision of a written action plan (WAP). However, many patients have poor asthma control and as few as 30 per cent have a WAP. Fabienne Djandji at the Saint-Justine University Central Hospital in Montreal, Canada, and co-workers conducted a survey of 421 doctors to determine their attitudes and provision of WAPs. Only 5.2 per cent of respondents provided WAPs to patients; those treating children or aiming for long-term asthma control were more likely to do so. The doctors said that incentives to provide WAPs would include requests from patients themselves, being paid to complete WAPs and having extra support from specialists or other health care professionals such as pharmacists.

## Introduction

Most patients with asthma have poor or suboptimal control of their disease with frequent symptoms, activity limitation, exacerbations, and health care resources consumption.^1, 2^ To maximise asthma control, international and national asthma guidelines recommend self-management asthma education, regular medical review, and the provision of a written action plan (WAP).^3–5^ A WAP is a personalised document that details how to maintain asthma control, what to do when losing control (i.e., when and what medication to increase or commence), and when to seek medical attention in case of loss of control.^3^ Strong evidence supports the beneficial effect on health outcomes of asthma education that included the provision of a WAP.^6, 7^ Importantly, randomised paediatric trials confirmed the individual contribution of WAP itself to increase treatment adherence, reduce exacerbations, and improve control of asthma, whether the WAP was used as an adjunct to asthma education,^8, 9^ or alone upon discharge from the emergency department.^10^ Yet, while patients have strongly endorsed their usefulness,^11, 12^ less than 30% of patients with asthma presenting to the acute care setting^13, 14^ or in general population surveys own a WAP^15, 16^.

To address this large care gap and increase the use of a WAP, successful implementation entails a higher provision of WAP by health care professionals and a better use of the WAP by patients and families with asthma. Most implementation trials have targeted patients and caregivers to promote the use of a WAP,^17^ with few aiming to facilitate the provision of a WAP by physicians.^17, 18^ Yet, multiple obstacles faced by physicians to provide a WAP have been identified: lack of time, lack of integration in the clinical routine, poor access to structured WAP templates, forgetfulness, lack of monetary compensation, and low perceived usefulness.^19–21^ To address the former three obstacles, a structured three-colour zone WAP was designed in triplicate, including the prescription, a chart copy, and the patient’s take-home plan, thus allowing simultaneous writing of all documents.^22^ The WAP combined with a prescription was shown to be effective in improving the quality of physician prescriptions, increasing patient adherence to prescriptions, and improving patient outcomes.^23^ Sponsored by the national institute of excellence in health and social services in Quebec, Canada, this WAP has been distributed freely to Quebec health care professionals since 2008. Unfortunately, availability and efficacy is generally insufficient to ensure implementation,^24^ as interventions shown effective in a trial setting may be difficult to implement in clinical practice.^25^ A successful implementation strategy must overcome the key barriers, facilitate behavioural changes, and offer acceptable, and effective implementation solutions.^26^ A novel approach to identify promising strategies consists in asking physicians to propose enablers perceived or experienced as effective and implementable in their own practice. Shown highly successful,^27^ this approach has lead to both important practice changes and improved health outcomes.^27, 28^ Along these lines, a number of enablers to facilitate the provision of the WAP were identified by physicians during individual qualitative interviews; the main ones pertained to clinic reorganisation of care and improved inter-professional asthma management.^29^

The main objective of this study was to ascertain physicians’ endorsement of promising enablers as identified by fellow physicians in the first phase of this research programme,^29^ to facilitate the provision of a WAP (Table 1). Secondary objectives aimed to identify the proportion of physicians reporting using a WAP and the determinants of the provision of this plan by physicians.

**Table 1:** Questions regarding physicians’ endorsement of proposed enablers to increase their use of a WAP

Were you aware that Canadian and International guidelines advise physicians to provide a written action plan to every patient with asthma and to review it at each visit?	Yes				No		
*Your colleagues have proposed the following solutions to facilitate the use of a written action plan*	Not interested at all				Very Interested		
To what extent would you be interested in attending a training session on how to complete and effectively explain a written action plan to your patients?	0	1	2	3	4	5	
*I am/would be more inclined to use a written action plan if:* ^a^	Would decrease my use of a WAP			No Change	Would increase my use of a WAP		
i have/had access to a format of the written action plan that enables me to complete it on the computer	**0**	**1**	**2**	**3**	**4**	**5**	**6**
the written action plan offered by the INESSS was modified	**0**	**1**	**2**	**3**	**4**	**5**	**6**
The written action plan is/was added to the medical chart of each patient with asthma before their medical visit	**0**	**1**	**2**	**3**	**4**	**5**	**6**
I receive/would receive a copy of the written action plan completed by the specialist/consultant (e.g., allergist, pulmonologist, etc.) who has seen my patient	**0**	**1**	**2**	**3**	**4**	**5**	**6**
My patient requests/would request it	**0**	**1**	**2**	**3**	**4**	**5**	**6**
The written action plan, that I have/ would have completed myself for my patient, is /was explained by a paramedical healthcare professional	**0**	**1**	**2**	**3**	**4**	**5**	**6**
Would you use a Written Action Plan more frequently if you would receive a financial supplement for every written action plan completed and provided to your patients?	**Yes**				**No**		
I am comfortable with the professional activities of pharmacists enabling them to:^b^	Strongly disagree				Strongly agree		
Prepare a written action plan for the patient according to my prescription:	0	1	2	3	4	5	
Suggest me a written action plan for my patient in the context of a pharmaceutical opinion	0	1	2	3	4	5	

^a^Responses of 5 or 6 on the 7-point Likert scale from 0 to 6 were deemed indicative of high endorsement

^b^Responses of 4 or 5 on the 6-point Likert scale from 0 to 6 were deemed indicative of high endorsement

## Results

**Fig. 1:**
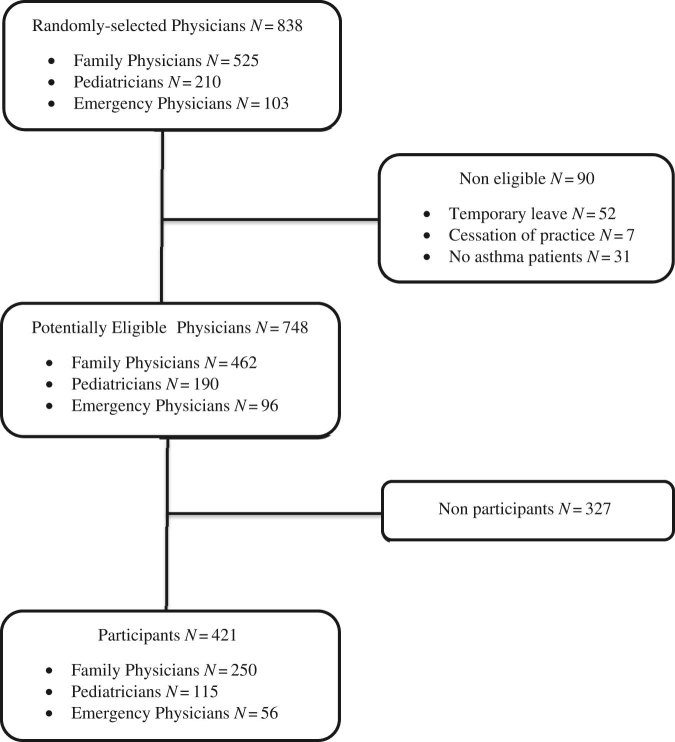
The flow of participants is depicted from screening to analysis

The survey was sent to 838 physicians, of whom 90 were found to be ineligible (retired, on leave or not seeing patients with asthma). Of the remaining 748 potentially eligible physicians, 421 (56%) physicians (250 family physicians, 115 paediatricians and 56 emergency physicians) returned the completed questionnaire (Fig. 1). Non-respondents were similar to respondents in their practice setting (rural vs. urban) and specialty, but differed significantly in sex (43% vs. 31% males, *P* < 0.001) and years of practice (20 vs. 13, *P* < 0.001). Respondents were predominantly female (69%), practicing in an urban (93%) and non-academic (56%) setting, and in practice for a median of 13 (5–21) years.

Only 43 (5.20%; 95% CI 2.71%, 7.68%) responders reported providing a WAP to 70% or more of their patients with asthma in their usual practice; they represented 27% of paediatricians, 4% of family physicians and less than 2% of emergency physicians. Approximately 60% of participants selected one of the acute care vignettes, with the remaining non-acute clinic vignettes equally distributed between the paediatric and adult cases.

Only 38.7% (32.8%, 44.5%) of physicians (61% of paediatricians, 38% of family physicians and 25% of emergency physicians) were aware of Canadian and international guidelines recommending the provision of a WAP to each patient. When notified of this recommendation, 58.7% (52.8%, 64.7%) of physicians (59% of family physicians, 49% of paediatricians and 39% of emergency physicians) expressed a high interest in attending a training session on how to efficiently complete and explain a WAP for patients.

**Fig. 2:**
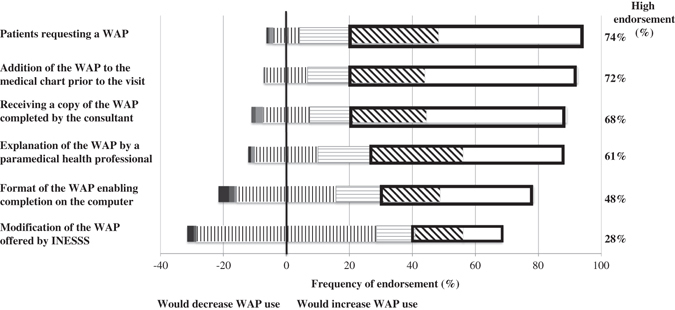
The graph depicts the proportion of physicians who reported, on a 7-point Likert-type scale from 0 to 6, whether the proposed enabler would change their use of a WAP. Answers 0 to 2 (would decrease use) represented by *black* (0), *dark grey* (1) and *pale grey* (2) *bars*; answer 3 (would not change use) is depicted by *vertical lines* displayed equally on either side of the central *vertical line*; and answers 4 to 6 (would increase use) are depicted by *horizontal lines* (4); *diagonal lines* (5), and *white bars* (6). The proportion of physicians responding 5 or 6, indicative of high endorsement of an experienced or anticipated increase in WAP use, is depicted in the bolded *rectangular box* and reported in the column on the right side, for each enabler

Of the seven most promising enablers,^29^ five that were highly endorsed by the majority of responders as likely to increase their WAP use were: patients requesting a WAP, the addition of a blank copy of the WAP to the medical chart prior to the medical visit, receiving a copy of the WAP completed by the specialist or consultant, and the explanation to the patient of the WAP completed by the physician by a paramedical health professional and not by the physician himself. In addition, 67.3% (61.7, 73.0) of physicians stated that a monetary incentive would increase their provision of a WAP. Access to a WAP version for completion by computer and modification of the existing WAP were the only two enablers that were not highly endorsed (Fig. 2).

A total of 126 (30%) physicians would have provided a WAP to the patient in the selected case vignette; they are hereafter considered as ‘intenders’. Overall, four determinants were strongly associated (Odds ratio, OR > 2.0) with the intention to use a WAP in the case vignette, namely the selection of a paediatric vignette, having long-term control as a treatment goal, being aware of national recommendations to provide a WAP to each patient with asthma to be reviewed at each visit, and being a paediatrician (Table 2), The latter three determinants held true for the subgroup of physicians who choose one of the two paediatric case vignettes, whereas female physicians and awareness of national recommendations were the two main determinants in those that selected an adult vignette. Emergency physicians were the lowest intended providers of WAP (OR, 0.31 95% CI 0.11, 0.93).

**Table 2:** Determinants of physician’s intention to use a written action plan (WAP) in the case vignette best representing their usual practice

	Intenders^a^(*N* = 126)	Non Intenders^a^(*N* = 294)	All cases	Paediatric Case Vignettes	Adult Case Vignettes
			Odd Ratios^b^ (95% CI)	Odd Ratios^b^ (95% CI)	Odd Ratios^b^ (95% CI)
Physician sex—n (%)					
Female	103 (81.8)	186 (63.3)			2.75 (1.05, 7.23)
Male	23 (18.3)	108 (36.7)			1
Case scenario^c^—n (%)					
Child	91 (72.2)	121 (41.2)	2.01 (1.10, 3.68)		2.40 (1.44, 5.02)
Adult	35 (27.8)	173 (58.8)	1		1
Awareness of recommendations to use WAP—n (%)					
Yes	81 (64.3)	97 (33.1)	2.94 (1.85, 4.76)	2.86 (1.56, 5.26)	3.23 (1.49, 7.14)
No	45 (35.7)	196 (66.9)	1	1	1
Treatment objective^c^—n (%)					
Improving long-term control	115 (91.3)	223 (75.9)	2.49 (1.20, 5.16)	3.27 (1.20, 8.96)	
Improving short-term control only	11 (8.7)	71 (24.2)	1	1	
Specialty—n (%)					
Pediatrics	64 (50.8)	51 (17.4)	2.09 (1.15, 3.89)	2.08 (1.10, 3.92)	–
Emergency Medicine	4 (3.2)	52 (17.7)	0.31 (0.11, 0.93)	0.32 (0.06, 1.56)	0.46 (0.09, 2.31)
Family medicine	58 (46.0)	191 (65.0)	1	1	1

Blank cells indicate that the variable was not statistically significant.

*WAP* written action plan

^a^Physicians who reported using a written action plan to the patient in their selected vignette were considered ‘Intenders’ in contrast to their counterparts, considered ‘Non Intenders’

^b^Odds ratio adjusted for speciality

^c^Regarding the patient in their selected case-vignette

## Discussion

### Main findings

In this group of randomly selected Quebec physicians, barely 5% reported routinely providing a WAP to their patients with asthma. About a third of them would have provided a WAP to the poorly controlled patient presented in the selected case vignette. To facilitate uptake of the WAP, physicians highly endorsed simple approaches to facilitate access to the WAP at the point of care, inter-professional management, the provision of monetary compensation, and patient request as key enablers to facilitate their provision of a WAP.

#### Interpretation of findings in relation to previously published work

Despite guidelines recommending its use for more than a decade,^3–5^ the proportion of physicians reporting regular use of a WAP for their patients with asthma remains abysmal. As the study did not focus on barriers but rather on potential solutions, the reason for such low use remains to be clarified. However, the intended use in the patient depicted in the case vignette was higher (30%), perhaps because of perceived higher need in view of their poor control. Indeed, physicians have consistently reported their selective use of WAP in patients they perceived more likely of benefitting because of more severe disease.^29^ The infrequent reported provision of WAP by physicians is concordant with the patient-reported WAP ownership rarely exceeding 30%, including among poorly controlled patients.^15, 16, 30, 31^ Even in the respiratory clinic of tertiary care centres, ownership of a WAP by asthmatic patients is often suboptimal at less than 50%.^11^

Of the seven enablers previously proposed by physicians in the first phase of this research programme,^29^ all but two were highly endorsed by physicians as likely to improve their delivery of WAP. Most physicians did not believe that a change in the template of the WAP, sponsored by the national institute of excellence in health and social services in Quebec, was required, perhaps due to the highly consultative approach to its development, the well-endorsed structured template, and the time saving by simultaneous writing of the patient’s take-home WAP, chart copy, and prescription.^22^ Access to a computerised version of the WAP was perceived as useful by just 45% of physicians, perhaps because of a low use of electronic medical records (EMR) at the time of this survey (not documented) or the inconvenience of having to print for patients both the prescription and coloured WAP version from the EMR (in absence of governmental-approved electronic transmission). This contrasts with the strikingly high-achieved provision rate (>93%) of WAP to children discharged from hospital, when it was integrated into the electronic medical record.^32^

In contrast, a simple practice organisation change, such as adding the WAP in the patient medical chart prior to the visit, was reported to be highly effective to increase physician’s personal use of the WAP. Consistent with the literature recognising the power of prompting on physician behaviour in general and for WAP in particular, a majority of respondents claimed that patient requests would markedly increase their provision of a WAP. Such an approach has been shown effective when the target behaviour has not been integrated into usual practice because of forgetfulness or inertia rather than non-agreement.^33^ Similarly, receiving the WAP completed by a specialist for a common patient suggests a domino effect of the positive example set by the specialist. Sending a copy of the WAP along with the consultation report and/or asking the patient to bring the WAP to the family physician are two readily implementable solutions. Indeed, the great majority of family physicians follow specialists’ recommendations and believe that consultants improve their own medical knowledge.^34^ With a lack of time reported as a key barrier to providing a WAP,^20^ the availability of a trained paramedical health professional (e.g., a nurse, respiratory technician or pharmacist) to explain the WAP to the patient, rather than the physician himself providing the explanation, was rated as a strong enabler, underlying the importance of inter-professional asthma management.^18^

Admitting their low awareness of guideline recommendations to provide or review the WAP at each visit, the overwhelming majority of physicians expressed a strong interest in attending a training session on how to efficiently complete and explain the WAP. These collective findings attest to the physicians’ general agreement with the four elements identified as likely to promote asthma plan delivery in Ring and colleagues’ theoretical model of action plan implementation, namely activities to support professional education, parent/carer education, partnership working, and communication.^18^ Finally, consistent with an American survey, in which half of physicians reported that the lack of a financial compensation was a barrier in providing adequate education to a patient newly diagnosed with asthma,^20^ most physicians indicated that a monetary incentive would positively affect their provision of a WAP, as shown in the Australia asthma 3+ visit plan.^35^ In several large health care systems, monetary incentives to pay physicians for their performance have shown increased efficiency and modest improvements in outcomes, but have raised issues regarding the validity of quality indicators on which to gauge performance, in this case WAP in paper or electronic version, and the added administrative requirements.^36^

Four determinants were strongly and independently linked to the physicians’ intention to provide a WAP. By far, the strongest determinant of the intention was the awareness of guideline recommendations, which was associated with a 3-fold higher odd of intention. Surprisingly and in contrast to other physician self-reports,^31^ the overwhelming majority of Quebec physicians caring for patients with asthma, primarily family physicians and emergency physicians, were unaware of the national and international recommendations to provide a WAP to each patient with asthma.^37^ Clearly, consensus guidelines have not adequately reached family physicians and emergency physicians. This reality calls for more effective strategies, such as delivering brief recommendations to clinicians via email.^38^ A novel finding was the strong association, independent from specialty, between the physician’s therapeutic objective to achieve long-term asthma control and the provision of a WAP, underlying the importance of disseminating this message to all physicians, irrespective of practice settings and specialty.

In line with previous reports,^15, 39, 40^ WAP was more frequently provided to children than adults in the case vignettes and more often used by paediatricians than by family physicians or emergency physicians. This begs the question as to whether the streetlight coloured WAP may be considered too childish for adults or whether written communication is perceived as less necessary in adults than children. While the effectiveness of asthma education including a WAP has been clearly established in both adults and children^6, 7, 41, 42^, the independent contribution of the WAP has only been demonstrated in children^23^ and is currently under review in adults,^43^ perhaps adding to the common, yet unproven, perception that these documents may not be useful or adapted for use by adults.

### Strengths and limitations of this study

The study must be interpreted in light of the following limitations. With a 56% participation rate, some of the analyses might have been underpowered, as we did not meet our target sample size. Also, we cannot rule out the possibility of a selection bias, as responders were more frequently women with fewer years of practice than non-responders and thus who might have been trained under more recent guidelines. This over representation of women and younger physicians selection bias is consistent with other studies done in the form of physician surveys.^44^ As for any survey, the findings represent reported intention, not observed practice. However, in view of the very low reported use of WAP, a social desirability bias is very unlikely. As we did not ask about reasons for non-use of the WAP, we cannot exclude the possibility that it may be due to perceived ineffectiveness or irrelevance to the physician clientele as frequently reported in qualitative studies.^21, 31^ Yet, the most highly endorsed enablers are targeting barriers faced by intended users rather than in non-intenders, suggesting a high receptivity of responders. Moreover, the high endorsement of enablers among all physicians, irrespective of their intended use or not of the WAP, suggests wide applicability.

The physicians we surveyed worked in a publicly funded health setting with free access to medical care, and where all patients are insured for their medication, either publically or privately. Asthma education centres with paramedical health care professionals and WAPs are readily available in Quebec caution is warranted before generalisation to other settings.

### Implications for future research, policy and practice

Highly endorsed enablers offer the means to increase WAP use by physicians. These include patient-prompting, consultants’ sharing with primary care providers of the WAP issued to their patients, organisational changes to facilitate WAP access at the point of care, delegating to paramedical healthcare professionals the explanation of the WAP, and financial incentives.

## Conclusion

In view of the very low reported use of the WAP by Quebec physicians, interventions to increase the awareness of guideline recommendations, of the importance of seeking long-term asthma control, and of the value of WAP in adults and in the acute care settings are likely to increase the intention to use WAP.

## Methods

This paper reports a survey of randomly selected physicians in the province of Quebec, Canada and represents the second phase of a mixed methods research programme. In the first phase of the study,^29^ qualitative semi-structured interviews were conducted in 42 physicians which identified 867 enablers of optimal asthma management including the use of WAP. Enablers were most frequently endorsed by interviewed physicians were reviewed using a 2-step Delphi approach by seven co-authors with different specialties to identify enablers most likely to be implementable for the questionnaire. The survey was then pretested in six physicians.

The research ethics board of the Sainte-Justine university health centre approved the study. The study was endorsed by the *I*nstitut national d’excellence en santé et services sociaux (INESSS), the Association des pédiatres du Québec, the Association des spécialistes en médecine d’urgence du Québec, and the Fédération des médecins omnipraticiens du Québec. Participants were notified by an information letter of study objectives; consent was assumed if they completed and returned the questionnaire.

Our survey methods have been described in details elsewhere.^45^ Briefly, participants were randomly selected from the list provided by the Collège des médecins du Québec*,* using a stratified sampling procedure based on specialty. Physicians were eligible if they were registered as family physicians, paediatricians, or emergency physicians in active practice in 2013 and reported seeing patients with asthma. As the INESSS has made available a WAP for asthma attacks since 2007, we specifically wished to include emergency physicians who often see patients during the initial exacerbation leading to the diagnosis of asthma and for subsequent exacerbations requiring health care consumption. Physicians who were retired, in training, or who had participated in the pre-test of the questionnaire or in phase-1 qualitative interviews^29^ were excluded and replaced.

The questionnaire was comprised of 102 questions. The first two sections included physician demographics and four case vignettes of a poorly controlled patient (a child or an adult presenting in a clinic or in an acute care setting) from which physicians chose the one vignette most closely representing their practice setting to anchor their treatment recommendations and means of communicating them to their patients. The next three sections pertained to beliefs, knowledge, and perceived facilitators regarding: the prescription of long-term inhaled corticosteroids; the provision of a WAP; and views on the expansion of pharmacists’ professional activities. The current article focuses on the 11 questions pertaining specifically on the WAP and seven promising enablers to increase WAP uptake in daily practice (Table 1).

Using the modified tailored design method*,* a written pre-notification letter was sent. Ten days later, we sent an information letter, the questionnaire, a copy of the WAP combined with a prescription,^22, 46^ a 25$ monetary incentive, and a pre-paid, pre-addressed return envelope. A reminder/thank you postal card was sent on day 21, followed by a second copy of the questionnaire on day 37 to the non-responders. Up to three phone calls were made to non-responders starting on day 38.

### Statistics

A sample size of 500 physicians was required to obtain a 95% confidence interval of ± 0.10 for endorsement proportions of 50%. Assuming a 60% response rate, we send the questionnaire to 838 physicians.

The distribution of endorsement of enablers was reported as the percentage (95% CI), after adjustment for the stratified sampling by specialty with greater weight given to the responses of family physicians (91%), vs. paediatricians (7.6%) and emergency physicians (1.4%) to reflect the actual distribution of these specialties in the province of Quebec. We classified physicians as being in strong agreement with promising enablers if they responded 5 or 6 on the 7-point scale (0 to 6) or 4 or 5 on a 6-point scale (0 to 5) or yes to direct question. We displayed endorsement of enablers with diverging stacked bars charts. We further explored potential determinants of the provision of a WAP to the patient described in the clinical vignette using bivariate and multivariate logistic regression models. Potential determinants included: physician demographics, practice characteristics, and responses to case vignettes. As sampling was stratified by specialty, specialty was forced into all models. All tests were two-sided with estimates presented with 95% confidence intervals. Analyses were performed on SAS^®^ software (version 9.3, SAS Institute Inc. Cary, NC 27513, USA). An OR with a 95% confidence interval between 0.9 and 1.1 was deemed indicative of equivalence. *P-*values less than 0.05 indicated statistical significance, with no correction for multiple testing.
